# Phage-Display Based Discovery and Characterization of Peptide Ligands against WDR5

**DOI:** 10.3390/molecules26051225

**Published:** 2021-02-25

**Authors:** Jiawen Cao, Tiantian Fan, Yanlian Li, Zhiyan Du, Lin Chen, Ying Wang, Xin Wang, Jingkang Shen, Xun Huang, Bing Xiong, Danyan Cao

**Affiliations:** 1 Department of College of Pharmacy, University of Chinese Academy of Sciences, No. 19A Yuquan Road, Beijing 100049, China; s18-caojiawen@simm.ac.cn (J.C.); 201628012342004@simm.ac.cn (T.F.); ylli@simm.ac.cn (Y.L.); irenedi1986@sina.com.cn (Z.D.); chenglinzd03@simm.ac.cn (L.C.); ying_qiqi@aliyun.com (Y.W.); wangxin@simm.ac.cn (X.W.); jkshen@simm.ac.cn (J.S.); xhuang@simm.ac.cn (X.H.); 2 Department of Medicinal Chemistry, Shanghai Institute of Materia Medica, Chinese Academy of Sciences, 555 Zuchongzhi Road, Shanghai 201203, China; 3 Division of Anti-Tumor Pharmacology, Shanghai Institute of Materia Medica, Chinese Academy of Sciences, 555 Zuchongzhi Road, Shanghai 201203, China

**Keywords:** phage display, biopanning, WDR5, cocrystal structure

## Abstract

WD40 is a ubiquitous domain presented in at least 361 human proteins and acts as scaffold to form protein complexes. Among them, WDR5 protein is an important mediator in several protein complexes to exert its functions in histone modification and chromatin remodeling. Therefore, it was considered as a promising epigenetic target involving in anti-cancer drug development. In view of the protein–protein interaction nature of WDR5, we initialized a campaign to discover new peptide-mimic inhibitors of WDR5. In current study, we utilized the phage display technique and screened with a disulfide-based cyclic peptide phage library. Five rounds of biopanning were performed and isolated clones were sequenced. By analyzing the sequences, total five peptides were synthesized for binding assay. The four peptides are shown to have the moderate binding affinity. Finally, the detailed binding interactions were revealed by solving a WDR5-peptide cocrystal structure.

## 1. Introduction

Scaffold proteins, acting as mediators through protein–protein interactions, are essential components in the formation of large protein complex structures. Among the most abundant protein interaction domains in the human proteome is the WD40 repeat (WDR) domain, which consists of at least 361 well annotated proteins [1,2]. The WDR domain typically folded into a donut shape with seven bladed β-propellers [3,4,5]. Among them, WDR5 has been recognized as an important binding partner in several chromatin modulating complexes such as COMPASS (Complex of Proteins Associated with SET1) complex that utilize the SET1/MLL protein to catalyze the di- and tri-methylation of histone H3 lysine 4 (H3K4) [6,7]. From these earliest studies, it was found that WDR5 also acts as a H3K4-methylation reader to recognize the substrate and facilitate the catalysis function of the whole complex. Recently, evidences indicated that WDR5 also participates in another complex called non-specific lethal (NSL) complex [8], in which WDR5 associated with KANSL1 or KANSL2 protein and MOF (Males absent On the First, a histone H4 acetyltransferase) to acetylate the histone H4 protein [9]. Another intriguing role of WDR5 is within the nucleosome remodeling and deacetylase (NuRD) complex, a chromatin-associated protein complex that performs the dual-roles of chromatin remodeling and histone deacetylation [10]. In this complex, WDR5 interacts with MBD3C protein to contribute to maintenance of the stem-like state for embryonic stem cells [11]. In 2015, Tansey’s lab reported [12] that WDR5 can be directly contacted by sequence-specific transcription factors by interacting with c-Myc protein, providing an illustrative example of another molecular function for WDR5.

Besides these physiological and biological roles of WDR5, it also plays important roles in sets of pathology development, especially in cancer. MLLs have been reported to associate with the overexpression of Homeobox (HOX) genes, which is frequently observed in acute leukemias such as acute lymphoblastic leukemia (ALL) and acute myeloid leukemia (AML) [13,14]. In 2015, Grebien et al. [15] reported a potent chemical probe OICR-9429 that binds to WDR5 at the same site (WIN site) for the interaction with MLL proteins, which could selectively inhibited proliferation and induced differentiation in p30-expressing human AML cells. More broadly, MYC protein, a newly identified WDR5 binding partner, is a notorious onco-protein of many types of cancer. Thomas et al. [16] performed the point mutation at the Myc binding site of WDR5 (WBM site) revealed that disrupt interaction with WDR5 attenuate binding of MYC to ~80% of its chromosomal locations and disable its ability to promote induced pluripotent stem cell formation and drive tumorigenesis. Based on these studies, WDR5 was considered as a novel drug target in oncology and attracted researchers worldwide to contribute the efforts. Over the past five years, there are many chemotype inhibitors have been reported to target two binding sites of WDR5. For the WIN site, except the previously mentioned OICR-9429, a series of 6,7-dihydro-5Hpyrrolo[1,2-a] imidazole compounds showed nanomolar binding activity as well as micromolar cellular activity against an AML-leukemia cell line [17]. Recently, Fesik et al. [18] reported a novel compound containing a dihydroisoquinolinone bicyclic core exhibits exceptional binding affinity. They also discovered a first series of biaryl sulfonamides compounds with low nanomolar affinity targeting the WBM site of WDR5.

Given the protein–protein interaction nature of WDR5 protein, we launched a campaign to identify peptide-mimic inhibitors of this protein. In the present study, we employed the phage display technology [19,20] to perform the biopanning experiments with a disulfide-based cyclic peptide phage library. In total, five rounds of biopanning were conducted and 122 isolated clones were sequenced. Four peptides were synthesized for binding assay and the interactions were confirmed with X-ray crystallography. This provided a starting point for further designing potent peptide-mimic inhibitors in WDR5 drug development.

## 2. Results and Discussion

### 2.1. Affinity Selection of Cyclic Peptides Specific to WDR5

A disulfide-constrained peptide phage display library (Ph.D.™-C7C Phage, CX_1_X_2_X_3_X_4_X_5_X_6_X_7_C.) from New England BioLabs was used in the biopanning experiment. The complexity of this library is up to 10^9^ independent clones which is thought to provide enough capacity to screen for the bioactive peptides against WDR5 target protein. According to the advantages of biopanning in solution, such as increasing the active surface between phage and the target and allowing a monovalent interaction, we used the His_6_-WDR5 as the target and immobilized it to Ni-NTA magnetic beads in solution.

To remove false-positive target-unrelated peptides (TUPs) which directly bind to Ni-NTA magnetic beads, the library was depleted 2 times by incubating the phage library directly with Ni-NTA magnetic beads before the biopanning against the target in the second and third rounds. Then In first three consecutive rounds of biopanning, the number of phages isolated out from the library is 1.2 × 10^7^ phage forming units (PFU), 7.5 × 10^7^ PFU, 9.5 × 10^8^ PFU in rounds 1, 2 and 3 respectively, (Table 1) counting by the phage tittering assay.

**Figure 1:**
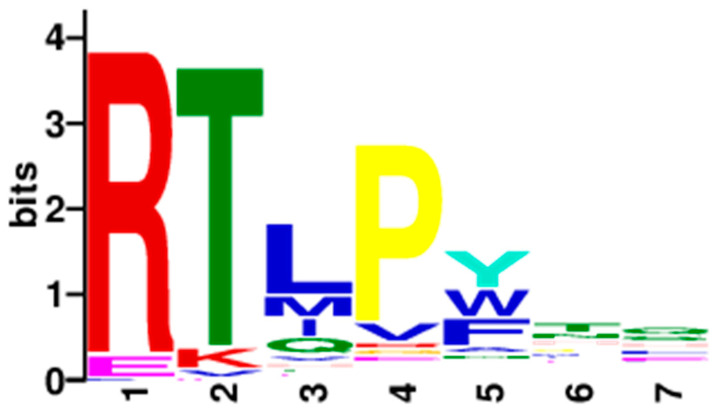
Random sequence comparison of the phage clone sequencing results of the third biopanning.

After the third round of biopanning, 80 clones were randomly selected for PCR reaction and Sanger sequencing (All the sequences are provided in Supplementary Information see Figure S1). Firstly, we found that there were multiple high-frequency sequences (Table 2), such as CRTLPFHEC(7/80), CEKMVATHC(4/80) and CRTLPWNQC(3/80). Regarding of this finding, we wanted to explore whether the higher frequent sequences have higher binding activity, so we synthesized the two sequences: CRTLPFHEC and CEKMVATHC. Besides, the sequencing result showed that most of the cyclic peptides contained “RT” consensus motif, and only 13 potential sequences were different without “RT” motif. Then an in-house Python software and MEME tools [22] were used to calculate the frequency of amino acids at each position, and the result was shown in Figure 1. Based on the third round of 80 sequencing data, it shows that except for the fixed cysteine at both ends, the first four variable amino acids are mainly “RTLP”. The fifth amino acid X_5_ is more likely to be tyrosine residue (26.6%), followed by tryptophan and phenylalanine (20.3% and 20.3% respectively). According to the classification of amino acids, tyrosine, tryptophan and phenylalanine are all aromatic amino acids containing a benzene ring structure. The amino acid sequence of the X_6_X_7_ motif is relatively random, and does not concentrate on some specific amino acids.

In addition, for better enrichment effect, another two rounds of bioppanning were carried out. Since there is no false-positive target-unrelated peptides in the sequencing of the third round biopanning, in the rounds 4 and 5 the eluted amplified phage library were depleted only one time. After the fifth round of biopanning, 42 clones were randomly selected for PCR reaction and Sanger sequencing. To our surprise, 24 cyclic peptides were found to contain multiple histidine amino acid motifs, which indicated that these histidine-enriched peptides may bind to Ni-NTA magnetic beads. Based on the result of other 18 sequences from the fifth rounds of sequencing, it was found that the pattern of the sequences is similar to the third round, with the predominant motif of “CRTLPY(W)NNC” (Supplementary Information see Figure S2). Detailed analysis of the sequencing data, we noticed that the first four residues (RTLP) is same to the sequencing data from third round, and the fifth position of the sequence is likely to be an aromatic residue, such as Y, W, and F. Based on the analysis, we synthesized another three sequences: CRTLPWNNC, CRTLPYGAC and CRTLPFGSC.

Among them, CRTLPWNNC is the consensus sequence derived from the sequence alignment (Supplementary Information see Figure S2). While others two sequences (CRTLPYGAC and CRTLPFGSC) are selected with the aim to assess the importance of the aromatic residues at the fifth position.

There are two protein–protein binding sites on WDR5: A shallow cleft on the side surface known as the “WDR5-binding motif” (WBM) site, and the other one located at the center of the protein, which referred to as the “WDR5-interacting” (WIN) site and usually bound with an arginine residue from its binding partners. Arginine is an essential amino acid for the binding as it inserts into the central WIN pocket of WDR5 and contributes important hydrogen bonding interactions. Based on the result derived from the third and fifth round of sequencing, it was found that the motif is mainly “RTLPY(W/F)XX”. Therefore, we guessed that we mainly screened the peptide ligands of the WIN site. To compare with the identified direct interacting partners of WDR5 [4], we collected the sequence segment at the binding interaction interface from these WDR5 binding partners. The aligned sequences in the Table 3 showed that the high-frequency consensus motif is “RT(S)EP”, while the consensus sequence of our sequencing results is “RTLPY(W/F)”. Clearly, the similarity exists in “RT(S)” motifs. However, residues at other position are quite different, especially at position X_5_, which in our identified sequences is mainly an aromatic amino acid (W/Y/F). This is consistent with the sequence bias such as Trp of phage display screening reported in the literature [23]. In the phage display procedure which is mainly affinity driven, the selected peptides may be over-hydrophobic and contain especially aromatic amino acids. In the research of targeting Erbin PDZ domain, Katja Luck and Gilles Travé observed a “super-binding peptide” with Trp at P1 position displaying high affinity which is robust against mutations at other peptide positions, it indicated a strong contribution of Trp to the binding affinity. Given the wide use of phage display for the determination of binding specificities of domain and peptide motif interactions, this leads us to guess that the preference of X_5_ amino acids to aromatic amino acid (W/Y/F) may be due to the tendency of phage display to select high affinity binders.

### 2.2. Measurement of Binding Affinity of Synthetic Peptides

**Figure 2:**
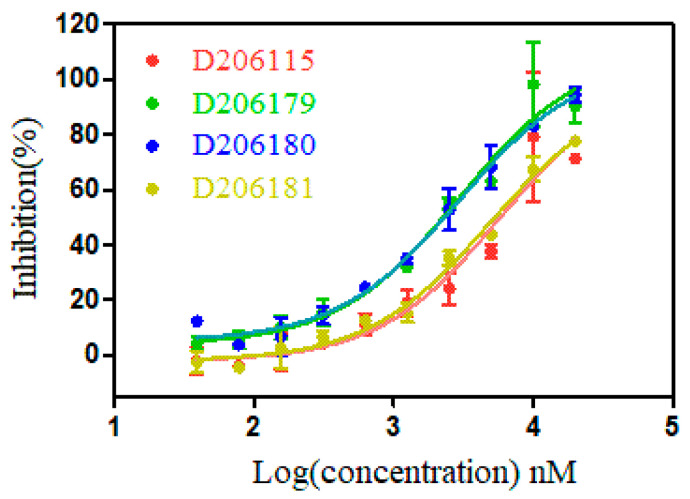
Results derived from FP direct competitive assays of 4 potential cyclic peptides targeting WDR5. Data points represent an average of two independent experiments; error bars are standard deviation.

Based on the results of sequencing, the frequency of sequence and the similarity with the WDR5 binding partners, we finally synthesized 5 cyclic peptide sequences: CRTLPFHEC(D206115), CEKMVATHC(D206116), CRTLPWNNC(D206179), CRTLPYGAC(D206180), and CRTLPFGSC(D206181) for further evaluation. The binding affinity of the synthetic cyclic peptides targeting WDR5 protein in vitro was obtained with a fluorescence polarization (FP) competitive assay [24]. Results from the direct FP binding assay were consistent with our expectation (Figure 2, Table 4). CEKMVATHC without “RT” motif was unable to bind to WDR5. The remaining sequences containing “RT” motifs can show certain binding activity, and we found that peptide CRTLPYGAC has the good binding activity with an EC_50_ of 2.7 ± 0.2 μM. Interestingly, the consensus sequence CRTLPWNNC derived from sequence alignment and scoring calculation showed the highest binding affinity among these five synthesized peptides (EC_50_: 2.3 ± 0.6 μM).

### 2.3. Binding Interactions from the Crystal Structure

**Figure 3:**
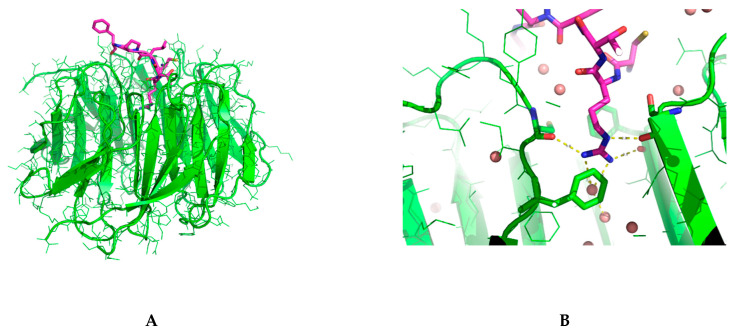
The cocrystal structure of D206115 with WDR5 (PDB code: 7DNO). (**A**) The overall complex structure. The WDR5 was shown in green cartoon mode and peptide in purple color. (**B**) The details of binding interactions between the arginine residue of D206115 and the WIN site of WDR5. Water molecules are shown in sphere.

To analyze the details of binding interaction, we solved the cocrystal structure of D206115 bound to WDR5 (Supplementary Information see Table S1). However, as showed in Figure 3, the supposed cyclized peptide was adopted a linear conformation, which indicated that the disulfide bond was broken. By scrutinizing all the reagents used in protein buffer and crystallization, we found that high concentration of Tris(2-carboxyethyl) phosphine (TCEP) was used to prevent the aggregation of WDR5 proteins during the crystallization. We tried to lower the concentration of TCEP and redo the crystallization but with no success. Nevertheless, the solved cocrystal structure still provided much information about the binding interactions between D206115 and WDR5. From Figure 3, we can realize that the arginine residue stretches into the deep pocket of WIN site, which formed extensive hydrogen bonding interactions with nearby residues and water molecules. Comparing to WDR5 bound with a peptide segment from MLL2 (PDB ID: 3UVK) [25], it was found that the arginine residue from MLL2 is located at the exact same position as in our solved structure, further indicating it is important for the binding interactions (Supplementary Information see Figure S4). Besides, the positive-charge nature of guanidyl group sandwiched between two aromatic residues Phe133 and Phe263, forming the typical cation-Pi interactions. Except the residue arginine in peptide D206115, other residues generally just situated at the top of the WIN binding site, without any visible direct interactions with WDR5 protein.

## 3. Materials and Methods

### 3.1. Bacterial Stains, Phage Library

The *Escherichia coli* ER2738 for the phage amplification and infection and the M13 phage library (Ph.D.™-C7C, cat. no. E8120S) screening kit both were obtained from New England Biolabs (Ipswich, MA, USA). All other reagents such as culture medium, bacterial antibiotics, buffer, etc used were of analytical grade and were purchased from Sigma (Sigma Aldrich, St. Louis, MO, USA).

### 3.2. Purified Protein for Biopanning and Crystallization

DNA fragment encoding amino acids 23 to 483 of WDR5 subcloned in the pET-28a plasmid was provided by Generay Co., Ltd. (Shanghai, Chia). The constructed plasmid containing six histidine or sumo tags in the N-terminus was transformed into *Escherichia coli* BL21(DE3) cell with heat shock. Then transformed *E. coli* cells were cultured at 37 °C in TB medium containing 50 mg/L kanamycin and the proteins was expressed by induction at an OD600 of 1.2–1.5 with 0.3 mM isopropyl β-d-1-thiogalactopyranoside (IPTG). After overnight induction, cells were harvested by centrifugation at 4000× *g* for 15 min at 4 °C and washed with 1× PBS twice. Then the cell pellets were resuspended in lysis buffer (50 mM Hepes pH 7.5, 250 mM NaCl, 2 mM imidazole, 5% (*v/v*) glycerol and 5 mM TCEP). Following cell lysis with an ultra-high pressure cell disrupter (UH-03, Union, Shanghai, China) and high-speed centrifugation at 11,500 rpm for 1 h at 4 °C, the insoluble pellets were collected. The supernatant was loaded onto a Ni-NTA column (GE Healthcare) washed twice with wash buffer containing 50 mM imidazole. The WDR5 protein was eluted with buffer containing 20 mM Hepes pH 7.5, 250 mM NaCl, 250 mM imidazole, 5 mM TCEP and 5% (*v/v*) glycerol then incubating with TEV protease overnight at 4 °C. The purified WDR5 protein was further purified by gel filtration chromatography (Superdex 200, GE Healthcare, Shanghai, China) with buffer containing 20 mM HEPES pH 7.5, 250 mM NaCl, 5 mM TCEP. Finally, the purified proteins without tags was concentrated to 18 mg/mL for crystallization experiments. Differently, the six histidine-tagged purified WDR5 protein was concentrated for biopanning without TEV protease cleavage.

### 3.3. Biopanning of Phage-Displayed Cyclic Peptide

Based on the previous selection methods for reference [26,27], we used a optimized panning strategies which performed in solution according to our own experimental purposes. For the first round of biopanning, the M13 Phage with 1 × 10^11^ phage forming units (pfu) was inputed and 2 × 10^11^ phage forming units (pfu) for the round 2, 3, 4, and 5. Six histidine-tagged protein WDR5 with the phage display library were incubated for overnight at 4 °C. Then the target protein (the final concentration in the system is 0.01μM) was immobilized on 100 μL of Pierce^TM^ Ni-NTA Magnetic Beads (Thermo Scientific, Waltham, MA, USA, cat. no. 78606), which was blocked with 1% bovine serum albumin (10% BSA, 90%TBS buffer) for 1 h at 4 °C and washed 4 times with washing buffer (PBS/Tween/imidazole: 1× PBS, 0.05% (*v/v*) Tween-20, 20 mM imidazole). The sample containing the target protein and phage incubate with the magnetic beads at 4 °C for 3 h with gentle vortexing. Then the beads were washed ten times with washing buffer (PBS/Tween/imidazole: 1× PBS, 0.05% (*v/v*) Tween 20, 20 mM imidazole). After washing, the bound phages were eluted by incubation with 1 mL of 0.2 M glycine, pH 2.2 (containing 1 mg/mL BSA) for 30 min on a rotating wheel at room temperature (RT). The eluted supernatant was separated with a magnet and were neutralized with 150 μL of 1 M Tris–HCl, pH 9.1 for 20 min at RT. Then it was used to infect *Escherichia coli* cells that amplified the phages. Subsequently, the overnight cultures of *Escherichia coli* ER2738 were diluted 1:100 and subcultured at 37 °C for 3.5 h. The eluted phages were transferred to 40 mL culture medium for further amplification. The titration and purification of the bound phage clones or amplified phages were performed according to the manufacturer’s protocol. The amplified eluates were used for the next biopanning round.

To remove binders to Ni-NTA Magnetic Beads, the library was depleted 2 times by incubating the phage library directly with magnetic beads for 2 h at 4 °C each time before selection against the target in the rounds 2 and 3. Differently, the amplified phage library were depleted 1 times in the rounds 4 and 5. Comparing with the sequencing results from third round and fifth round, it suggested that TUPs relevant to Ni-NTA magnetic beads need to be depleted at least two times to overcome the preponderance of these histidine containing peptides.

### 3.4. Sanger Sequencing

After the successive rounds of biopanning, some blue single phage clones were randomly picked from the phage titer plates for sanger sequencing. Before sequencing, we used each single phage clone as a DNA template to perform a PCR reaction to reduce the difficulty of sequencing. The primers for the PCR amplification reactions were listed below. A 50 μL PCR reaction by adding reagents in the following order to achieve the final concentrations: 21 μL H_2_O, 25 μL 2×Hieff^®^Robust PCR Master Mix With Dye (cat. no.10106ES03*; Yeasen, Shanghai, China), 1 μL the forward primer, 2 μL the reverse primer, and the single plaque as the template.

All of the primers were 10 μM. Considering that the phage contained single-stranded DNA, which was different from the normal double-stranded DNA template, the reverse primer needed to be added twice as much as the forward primer to ensure the priority synthesis of double-stranded DNA before completing the subsequent PCR reaction. 30 PCR cycles were performed (95 °C for 15 s, 53 °C for 15 s and 72 °C for 30 s). The target band size of the product assessed by 1.5% agarose gel electrophoresis was 313 bp. Then the PCR product were sequenced by Personalbio, Inc., with Sanger sequencing. Subsequently, the amino acid sequences of the C7C cyclic peptides displayed on the phage were analyzed using DNAStar software (Lasergene7, DNASTAR, Inc., Wisconsin, WI, USA).

**Primer for PCR reaction:**
Forward C7C-1: 5′-GTCGGCGCAACTATCGGTATC-3′
Reverse C7C-R1: 5′-GCCCTCATAGTTAGCGTAACG-3′

### 3.5. Synthetic Peptides and Fluorescence Polarization (FP) Binding Assays

A total of 6 cyclic peptide compounds were synthesized by Dentripharm Co., Ltd. (Hangzhou, China). They were synthesized by solid phase synthesis. Firstly, the C-terminal amino acid was fixed on the resin carrier, and the subsequent amino acids were condensed to form active ester by condensation reagent to extend the amino acid peptide chain one by one. The peptide chains were cleaved from the resin using the trifluoroacetic acid to obtain the crude peptide. Then they were separated and purified using preparative high-performance liquid chromatography (Pre-HPLC, Agilent, Inc., Santa Clara, CA 95051, USA) and purified products were lyophilized for storing. The purity of the peptides was analyzed by the mass spectrometer (Bruker Daltonics micro TOF-Q, Hamburg, Germany) as well as analytical HPLC (Agilent 1100, Agilent, Inc., Santa Clara, CA 95051, USA). See Supplementary Information for more details (Figure S3).

The fluorescence polarization (FP) binding assays used to evaluate the ability of these cyclic peptides targeting protein WDR5 were carried out in the 384-well plate. We synthesized the fluorescently labeled positive compound [28,29]: 1-((3′,6′-dihydroxy-3-oxo-3H-spiro[isobenzofuran-1,9′-xanthen]-5-yl)amino)-*N*-(3′-(3,5-dimethylbenzamido)-4′-(4-methylpiperazin-1-yl)-[1,1′-biphenyl]-4-yl)-1-thioxo-5,8,11-trioxa-2-azatetradecan-14-amide, which was used in direct binding assay against WDR5. 10 μL of 10 nM positive compound was added to 20 μL WDR5(the final concentration was 5 nM) in the buffer containing 0.2 M phosphate; 300 mM NaCl; 0.1%CHAPS; PH 6.5; 0.5 mM TCEP. Then the peptide compound to be tested was diluted in a gradient and added to each well with 10μL. Finally, the assay was carried out in 40 μL volume and the protein tracer mixture was incubated for 4 h before reading the plate. The fluorescence polarization was measured in Synergy™ Neo2 Multi-Mode Microplate Reader using an excitation filter at 485 nm and an emission filter at 535 nm. Each experiment was done two times and each polarization readings consisted of two averaged measurements. The reading is processed by the following formula:

(1) $$\mathrm{Inhibition}=\frac{C-F}{C-B}\times 100\%$$

where C is the anisotropy value of the fluorescence substrate bound to the protein completely, B is the anisotropy value of the fluorescence substrate, F is the corresponding anisotropy value after adding different concentrations of peptides. The data were plotted against the log peptide concentration and was fitted into a one site-Fit logIC50 equation with Graphpad Prim5 software using nonlinear regression model to obtain the competitive curve:

(2) Y=A+B−A1+10^(x−LogIC50)

where x is log10 of the compound concentration in M, Y is the inflection point (EC50 or IC50), A is the bottom plateau effect, B is the top plateau effect. Potency (IC50/EC50) is the log of the concentration of competitor that results in binding half-way between bottom and top.

The EC50 values of peptides were determined in competitive binding experiments by nonlinear regression fitting of the competition curves as mentioned above. And Ki values of competitive inhibitors were obtained directly by nonlinear regression fitting, based upon the Kd values of the probe to different proteins and concentrations of the proteins and probes in the competitive assays [30]. The data is processed by the following formula:

(3) $$F_{0}=\frac{P+L_{0}+\mathrm{Kd}-\sqrt{{\left(P+L_{0}+\mathrm{Kd}\right)}^{2}-4\times P\times L_{0}}}{2\times L_{0}}$$

(4) $$\frac{{\mathrm{IC}}_{50}}{\frac{F_{0}\times \mathrm{Kd}}{\left(1-F_{0}\right)\left(2-F_{0}\right)}+F_{0}\times \frac{L_{0}}{2}}=\frac{\mathrm{Ki}\left(2-F_{0}\right)}{\mathrm{Kd}\times F_{0}}+1$$

where P is the proteins concentration in the assay (5 nM), L_0_ is the Tracer concentration in the assay (2.5 nM), F_0_ and the corresponding Ki value can be calculated according to the above formula.

### 3.6. Crystallization and Cocrystal Structure Determination

The purified proteins were concentrated to 18 mg/mL before crystal growth. The peptide was 500 mM by dissolving the compound in DMSO and added to the concentrated protein with incubating overnight at 4 °C. Crystallization was performed at 16 °C by the hanging-drop vapor-diffusion technique. Crystals were obtained by mixing 1.5 µL of the protein solution with 1.5 µL of a reservoir solution; the mixture drop was equilibrated against 500 µL of the reservoir solution containing 20–30% PEG3350 gradient dilution, 0.1 M Bris Tris, pH = 6.0, 0.2 M ammonium acetate (AsAc), H_2_O. And another 500 µL of the reservoir solution containing 22–33% PEG3350 gradient dilution, 0.1 M Hepes, pH = 7.5, 0.05 M (NH_4_)_2_SO_4_, H_2_O. After nearly 7 days, some crystals were discovered through a microscope under such conditions 20–30% PEG3350 gradient dilution, 0.1 M Bris Tris, pH = 6.0, 0.2 M AsAc, H_2_O.

Data were collected at 100 K on beam line BL17U at the Shanghai Synchrotron Radiation Facility (SSRF) (Shanghai, China) for the co-crystallized structures [31,32]. The data were processed with the HKL2000 [33] software packages (HKL Research, Inc., Charlottesville, VA, USA), and the structures were then solved by molecular replacement using the CCP4 program MOLREP [34]. The search model used for the crystals was the WDR5-MLL3 complex structure (PDB code: 3UVL). The structures were refined using the CCP4 program REFMAC5 combined with the simulated annealing protocol implemented in the program PHENIX [35]. With the aid of the program Coot [36], compound, water molecules, and others were fitted into the initial Fo—Fc maps.

## 4. Conclusions

**Figure 4:**
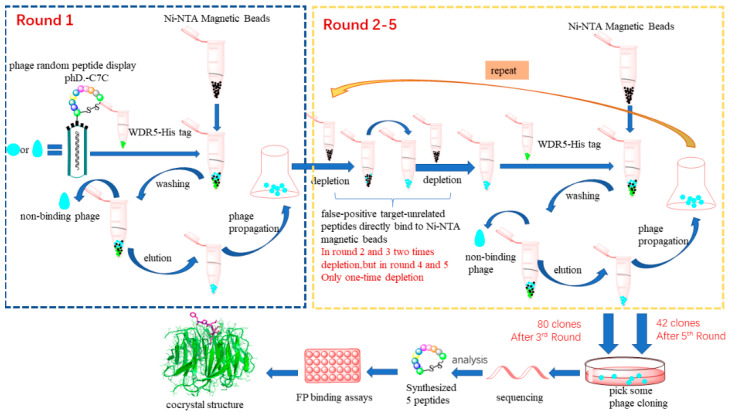
The flowchart of the whole phage display screening process.

WDR5, a promising epigenetic target in anticancer drug development, was selected for identify new peptide inhibitors with phage display technique. The whole process is illustrated as a flowchart shown in Figure 4.

Detailedly, to restrain the enormous conformations associated with linear peptides, we applied a disulfide-based cyclic peptide phage library containing 7 randomized residues expressed in M13 phage PIII protein. After the third round of biopanning, 80 isolated clones were selected for sequencing, and based on the resulted sequences, the consensus motif was discovered containing “RTLPW(Y/F)”. Another two rounds of biopanning were further conducted. The sequencing result was similar except many false-positive target-unrelated peptides containing multiple histidine amino acids were identified, which is due to in round 4 and 5 the eluted amplified phage library was depleted only one time while in round 1–3 two times depletion were performed. This implied that in the future biopanning study, at least two times depletion against Ni-NTA magnetic beads need to perform to remove the false positive sequences. Combined two sequencing data, five peptides were synthesized for testing the binding affinity, in which four peptides were shown to have moderate binding activities at μM level towards WDR5 WIN pocket. Finally, to reveal the details of binding interaction, we solved a cocrystal structure of WDR5 bound with peptide D206115. The essential binding interactions between the important residue arginine and the WIN binding site of WDR5 were illustrated, further reinforcement of the importance of arginine residue. Together, this study not only demonstrated that the phage display is a valuable technique to discover peptide modulators for WD40 proteins, it also provided two peptides as starting points for developing peptide-mimic WDR5 inhibitors.

## Figures and Tables

**Table 1:** Recovery efficiency after 5 rounds of biopanning. The recovery efficiency of each round was counted as the output divided by the input titer [21]. PFU, phage forming units.

	Input Titer (PFU)	Output Titer (PFU)	Recovery Efficiency	Fold Increase
Round 1	1.0 × 10^11^	1.2 × 10^7^	1.2 × 10^−4^	1
Round 2	2.0 × 10^11^	7.5 × 10^7^	3.8 × 10^−4^	3.1
Round 3	2.0 × 10^11^	9.5 × 10^8^	4.8 × 10^−3^	39.6
Round 4	2.0 × 10^11^	2.3 × 10^9^	1.2 × 10^−2^	95.8
Round 5	2.0 × 10^11^	2.3 × 10^9^	1.2 × 10^−2^	95.8

**Table 2:** Peptide sequences derived from the phage clones eluted after the third round of panning. The conserved similar and homologous amino acid residues between peptide sequences are marked with same colors. The frequency appears from high to low. The remaining parts are detailed in the appendix.

Round 3	Reads (Sanger)
CRTLPFHEC	7/80
CEKMVATHC	4/80
CRTLPWNQC	3/80
CRTIPFTHC	2/80
CRTMEYTSC	2/80
CRTLPYHLC	2/80
CRTQPYNQC	1/80

**Table 3:** Characterized Win motifs.

Partner	Motifs
MLL1	GSAR**A**EV
MLL2	GCAR**S**EP
MLL3	GCAR**S**EP
MLL4	GAAR**A**EV
SET1A	GSAR**S**EG
SET1B	GCAR**S**EG
H3	——AR**T**KQ
KANSL1	VAAR**T**RP
MBD3C	GAAR**C**RV
KIF2A	GSAR**A**RP

**Table 4:** Data from direct FP binding experiments; N.D.: not determined; The results are presented as mean ± standard error.

Targeting WDR5	EC_50_ (μM)	Ki (μM)
D206115-CRTLPFHEC	5.9 ± 0.2	1.2 ± 0.04
D206116-CEKMVATHC	N.D.	N.D.
D206179-CRTLPWNNC	2.3 ± 0.6	0.5 ± 0.1
D206180-CRTLPYGAC	2.7 ± 0.2	0.5 ± 0.05
D206181-CRTLPFGSC	4.4 ± 0.4	0.9 ± 0.08

## Data Availability

All data, models, and code generated or used during the study appear in the submitted article.

## Supplementary Materials

Supplementary Materials can be found online. It includes the details of sequencing data, the spectra data of synthesized peptides and the crystallography information of the cocrystal structure.

## Sample Availability

Samples of the compounds are available from the authors.
