# Supplementary material for: Phage-Display Based Discovery and Characterization of Peptide Ligands against WDR5
Source: Molecules. 2021 Feb 25;26(5):1225. doi: 10.3390/molecules26051225 (PMC7956166; doi:10.3390/molecules26051225)
Supplement: Supplementary file 1 [file molecules-26-01225-s001.pdf]

molecules

Article（Supporting information）

Phage-display Based discovery and Characterization of Peptide Ligands against WDR5

Jiawen Cao[1,2], Tiantian Fan[1,2], Yanlian Li[2], Zhiyan Du[2], Lin Chen[2], Ying Wang[2], Xin Wang[2], Jingkang Shen[2], Xun Huang[3], Bing Xiong[2,*], Danyan Cao[2,*]

[1] Department of Medicinal Chemistry, Shanghai Institute of Materia Medica, Chinese Academy of Sciences, 555 Zuchongzhi Road, Shanghai 201203, China; University of Chinese Academy of Sciences, No. 19A Yuquan Road, Beijing 100049, China

[2] Department of Medicinal Chemistry, Shanghai Institute of Materia Medica, Chinese Academy of Sciences, 555 Zuchongzhi Road, Shanghai 201203, China

* Division of Anti-Tumor Pharmacology, Shanghai Institute of Materia Medica, Chinese Academy of Sciences, 555 Zuchongzhi Road, Shanghai 201203, China; University of Chinese Academy of Sciences, No. 19A Yuquan Road, Beijing 100049, China

Received: 19 December 2020; Accepted: 21 February 2021; Published: 25 February 2021

Table of Content

1） **Figure S1. Sequencing data**

2） **Figure S2.The phage clone sequencing results of the fifth bopanning**

3） **Figure S3. HPLC and MS spectra of synthesized peptides**

4） **Table S1. Crystallography Table**

5） **Figure S4. Cocrystal structure comparison**

A

1	CRTMLLEDC	41	CEKMVATHC
2	CFT FL . EWC	42	CRT L PFHEC
3	CRTLPYQG C	43	CRTV PYQLC
4	CRTQDWRAC	44	CRTLPWAGC
5	CRTQP Y LYC	45	CRT I P F THC
6	CRTLPWNQC	46	CPL S P HNIC
7	CRTEVH STC	47	CRT L P IGHC
8	CRTI P Y SYC	48	CLNR I NLSC
9	CFTF I N GSC	49	CRVMPYPHC
10	CRTI P Y NNC	50	CEKMVATHC
11	CRTHP F PL C	51	CRT L PFHEC
12	CFPGLE EWC	52	CRTMPYAQC
13	CRTLPF H EC	53	CRT LPYS SC
14	CRTLPWNQC	54	CRT LPLN SC
15	CRTVPWEQC	55	CRTQ PF LYC
16	CRT I P FTHC	56	CRTLPWTNC
17	CRTQP F PNC	57	CRTLPWDKC
18	CRTLP I G PC	58	CQRTDGFKC
19	CRTMPYGFC	59	CRTHP F PSC
20	CRTLPYMQC	60	CEKMVSTHC
21	CVEMKPGHC	61	CRT I P FTKC
22	CRTLPYH LC	62	CRT L P FHEC
23	CRTLPWNQC	63	CRT L P FERC
24	CRTI PWYVC	64	CRT I PWTSC
25	CRTLPF H EC	65	CRTH PNMAC
26	CRDARWVQC	66	CRT LGYE LC
27	CRTQPHMS C	67	CRT LNNLAC
28	CRTTSWDF C	68	CRT LTMQAC
29	CRTIRT N MC	69	CTA TWTKVC
30	CRTLPYH LC	70	CRTMEYT SC
31	CRTLPYL QC	71	CRT LP FH EC
32	CRTQPYNQC	72	CRT LP FH EC
33	CEKMVATHC	73	CRT LPY F EC
34	CRTLPMNQC	74	CRTT PWS AC
35	CEKMVATHC	75	CRT I PWN LC
36	CRTQPYPWC	76	CRTV PYM SC
37	CRVQPWPQC	77	CTNWPP T VC
38	CRTLGVEC L	78	CRT L PVD LC
39	CRTLPFT G C	79	CRT E PYL NC
40	CRTLPWGI C	80	CRTM EYT SC

B

1	CKHGHHHWC
2	CSHHGHN HC
3	CHHAHHH SC
4	CR TAPNMNC
5	CRTMP FL HC
6	CGS IGEM NC
7	CRT LPWS AC
8	CSHHGHNHC
9	CHRHGHSHC
10	CHAHHHSSC
11	CRTLPWNQC
12	CRTQPLGHC
13	CIRHHHTHC
14	CNHKNHHHC
15	CRTLPYQGC
16	CHCHHHTHC
17	CHNHHHPFC
18	CTHHQSHHC
19	CHHHRHSDC
20	CHSHHHKDC
21	CTHHQSHHC
22	CRDSLSLHC
23	CRTQPYNQC
24	CHHHNHHRC
25	CNTHHRHHC
26	CRTMPWPFC
27	CRTVPWNNC
28	CSHHGHNHC
29	CRTL P YNGC
30	CTHHQSHHC
31	CWEGRVHNC
32	CINHHHHMC
33	CTHHQSHHC
34	CRTL P FGSC
35	CRTQP FLAC
36	CTHHQSHHC
37	CRTMPWGLC
38	CHHGSHHHC
39	CSHHGHNHC
40	CRTL P YGAC
41	CRTMPYQ LC
42	CTHHHKSHC

Figure S1. A) 80 phages-displayed cyclic peptide sequences from the third round of panning. B) 42 phages-displayed cyclic peptide sequences from the fifth round of panning.

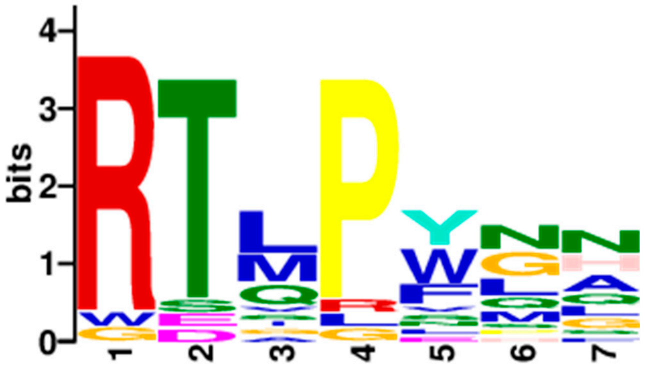

Figure S2. The phage clone sequencing results of the fifth bopanning.

Synthetic information of peptide D206115

A

Peptide Name: Custom Peptide

Purchase order number: D206115

Lot Number: FL-08-0115

Sequence: H-Cys-Arg-Thr-Leu-Pro-Phe-His-Glu-Cys-OH (Cys&Cys bridge)

Molecular Weight (daltons): 1103.3

Final Purity (HPLC%): 95.5%

Amount Supplied: 5mg

Appearance: White lyophilized powder

Storage Conditions: Long Term: -20°C; Short Term: 2°C ~ 8°C

Sample information

Sample Name: D206115/FL-08-0115
Sample Type: Unknown
Sample Bottle: 26
Number of Injections: 1
Injection Volume: 15.00 ul
Running Time: 25.0 Minutes

Collector: System
Sample Group Name: A20090701
Collection Method Group: 15% _ 45% _ B _ 08 _ 215 _ method group
Approach: M20090702
Channel Name: DAD.0.0
Processing Channel Description: DAD: Signal A, 215nm/ broadband: 4nm

Acquisition Time: 2020/9/7 12:56:59 CST
Processing Time: 2020/9/7 13:25:47 CST

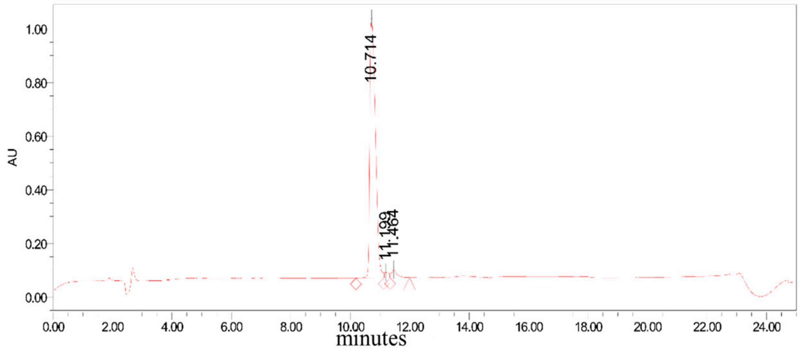

Peak Results

Name	Retention Time(minute)	%Area	Area(Microvolt*second)	Height(Microvolt)
1	10.714	95.52	12876617	967724
2	11.199	1.59	214838	20370
3	11.464	2.88	388609	30139

Mass Spectrum SmartFormula Report

Analysis Info

Analysis Name D:\Data\ESI-Luh-200910-D206115_01.d
Method ESI+100-800-200910.m
Sample Name
Comment

Acquisition Date 9/10/2020 3:56:44 PM
Operator BDAL@DE
Instrument / Ser# microTOF-Q II 228888.10324

Acquisition Parameter

Source Type	ESI	Ion Polarity	Positive	Set Nebulizer	1.2 Bar
Focus	Active	Set Capillary	4800 V	Set Dry Heater	220 °C
Scan Begin	100 m/z	Set End Plate Offset	-500 V	Set Dry Gas	3.5 l/min
Scan End	2000 m/z	Set Collision Cell RF	200.0 Vpp	Set Divert Valve	Source

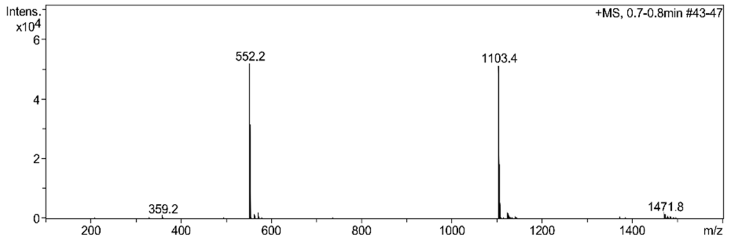

Synthetic information of peptide D206116

B

Peptide Name：Custom Peptide

Purchase order number：D206116

Lot Number：FL-08-0116

Sequence：H-Cys-Glu-Lys-Met-Val-Ala-Thr-His-Cys-OH（Cys&Cys bridge）

Molecular Weight（daltons）：1019.2

Final Purity（HPLC%）：95.3%

Amount Supplied：5mg

Appearance：White lyophilized powder

Storage Conditions： Long Term：-20°C；Short Term：2°C ~ 8°C

Sample information

Sample Name：D206116/FL-08-0116
Sample Type：Unknown
Sample Bottle：25
Number of Injections：1
Injection Volume：20.00 ul
Running Time：20.0 Minutes

Collector：System
Sample Group Name：A20090701
Collection Method Group：10 _ 40 B 07 _ method group
Approach：M20090701
Channel Name：DAD.0.0
Processing Channel Description：DAD： Signal A，220nm/ broadband：4nm

Acquisition Time：2020/9/7 9:48:30 CST
Processing Time：2020/9/7 10:16:22 CST

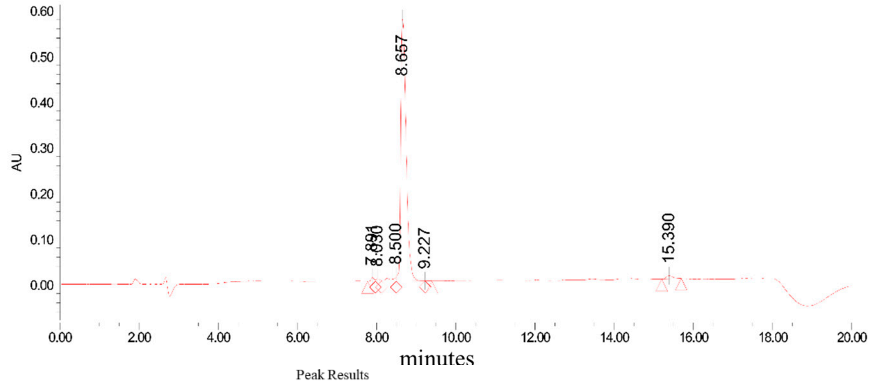

Peak Results

Name	Retention Time(minute)	%Area	Area(Microvolt*second)	Height(Microvolt)
1	7.891	0.77	44053	8585
2	8.030	0.60	34259	5825
3	8.500	1.81	103077	10494
4	8.657	95.35	5444349	574761
5	9.227	0.04	2153	611
6	15.390	1.44	82050	6740

Mass Spectrum SmartFormula Report

Analysis Info

Analysis Name D:\Data\ESI-Luh-200910-D206116_01.d
Method ESI+100-800-200910.m
Sample Name
Comment

Acquisition Date 9/10/2020 3:45:29 PM

Operator BDAL@DE
Instrument / Ser# microTOF-Q II 228888.10324

Acquisition Parameter

Source Type	ESI	Ion Polarity	Positive	Set Nebulizer	1.2 Bar
Focus	Active	Set Capillary	4800 V	Set Dry Heater	220 °C
Scan Begin	100 m/z	Set End Plate Offset	-500 V	Set Dry Gas	3.5 l/min
Scan End	2000 m/z	Set Collision Cell RF	200.0 Vpp	Set Divert Valve	Source

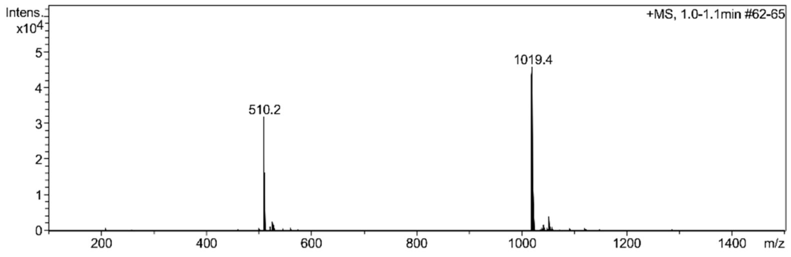

Synthetic information of peptide D206179

C

Peptide Name: Custom Peptide

Purchase order number: D206179

Lot Number: FL-10-0039

Sequence: H-Cys-Arg-Thr-Leu-Pro-Trp-Asn-Asn-Cys-OH (Cys&Cys bridge)

Molecular Weight (daltons) : 1104.3

Final Purity (HPLC%) : 97.6%

Amount Supplied: 5mg

Appearance: White lyophilized powder

Storage Conditions: Long Term: -20°C; Short Term: 2°C ~ 8°C

Sample information

Sample Name: D206179/FL-10-0039
Sample Type : Unknown
Sample Bottle: 1
Number of Injections: 1
Injection Volume: 10.00 ul
Running Time: 20.0 Minutes

Collector: System
Sample Group Name: A20102602
Collection Method Group: 20 _ 50 B _ method group
Approach: M20102602
Channel Name: DAD.0.0
Processing Channel Description: DAD: Signal A, 215nm/ broadband: 4nm

Acquisition Time: 2020/10/26 12:46:44 CST
Processing Time: 2020/10/26 13:43:37 CST

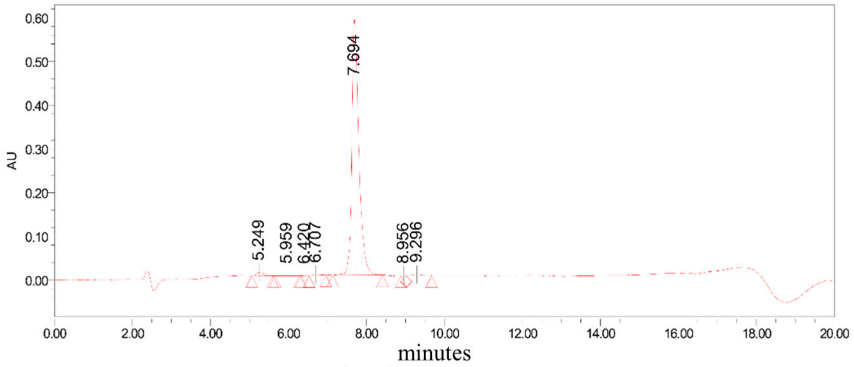

Peak Results

Name	Retention Time(minute)	%Area	Area(Microvolt*second)	Height(Microvolt)
1	5.249	1.20	90347	7413
2	5.959	0.20	15414	1087
3	6.420	0.03	2268	307
4	6.707	0.25	19164	1739
5	7.694	97.68	7382491	585540
6	8.956	0.02	1678	387
7	9296	0.61	46214	2956

Mass Spectrum SmartFormula Report

Analysis Info

Analysis Name D:\Data\ESI-LUH-201026-D206179_01.d
Method lcms-5-55.m
Sample Name
Comment

Acquisition Date 10/26/2020 3:57:27 PM
Operator BDAL@DE
Instrument / Ser# microTOF-Q II 228888.10324

Acquisition Parameter

Source Type	ESI	Ion Polarity	Positive	Set Nebulizer	1.8 Bar
Focus	Active	Set Capillary	4800 V	Set Dry Heater	220 °C
Scan Begin	100 m/z	Set End Plate Offset	-500 V	Set Dry Gas	6.0 l/min
Scan End	2000 m/z	Set Collision Cell RF	200.0 Vpp	Set Divert Valve	Waste

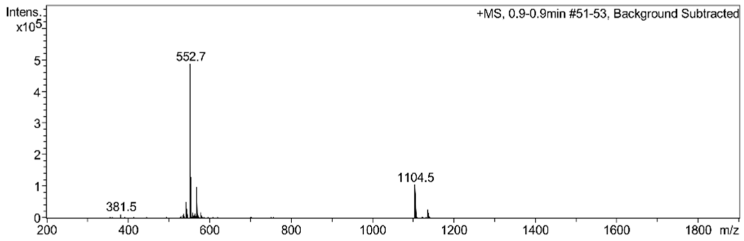

Synthetic information of peptide D206180

D

Peptide Name: Custom Peptide

Purchase order number: D206180

Lot Number: FL-10-0040

Sequence: H-Cys-Arg-Thr-Leu-Pro-Tyr-Gly-Ala-Cys-OH (Cys&Cys bridge)

Molecular Weight (daltons) : 981.2

Final Purity (HPLC%) : 99.6%

Amount Supplied: 5mg

Appearance: White lyophilized powder

Storage Conditions: Long Term: -20°C; Short Term: 2°C ~ 8°C

Sample information

Sample Name: D206180/FL-10-0040
Sample Type : Unknown
Sample Bottle: 4
Number of Injections: 1
Injection Volume: 10.00 ul
Running Time: 20.0 Minutes

Collector: System
Sample Group Name: A20102701
Collection Method Group: 10 _ 40 B 07 _ method group
Approach: M20102701
Channel Name: DAD.0.0
Processing Channel Description: DAD: Signal A, 220nm/ broadband: 4nm

Acquisition Time: 2020/10/27 9:12:30 CST
Processing Time: 2020/10/27 9:35:57 CST

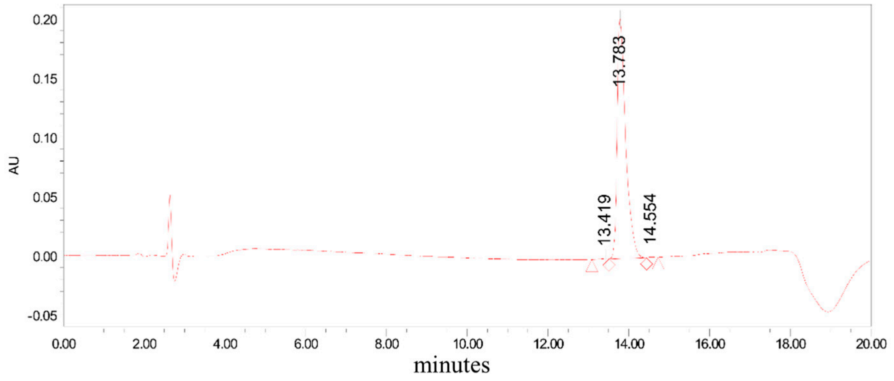

Peak Results

Name	Retention Time(minute)	%Area	Area(Microvolt*second)	Height(Microvolt)
1	13.419	0.25	7550	589
2	13.783	99.61	2996771	202424
3	14.554	0.14	4183	380

Mass Spectrum SmartFormula Report

Analysis Info

Analysis Name	D:\Data\ESI-LUH-201026-D206180_01.d	Acquisition Date	10/26/2020 3:56:13 PM
Method	lcms-5-55.m	Operator	BDAL@DE
Sample Name		Instrument / Ser#	microTOF-Q II 228888.10324
Comment			

Acquisition Parameter

Source Type	ESI	Ion Polarity	Positive	Set Nebulizer	1.8 Bar
Focus	Active	Set Capillary	4800 V	Set Dry Heater	220 °C
Scan Begin	100 m/z	Set End Plate Offset	-500 V	Set Dry Gas	6.0 l/min
Scan End	2000 m/z	Set Collision Cell RF	200.0 Vpp	Set Divert Valve	Waste

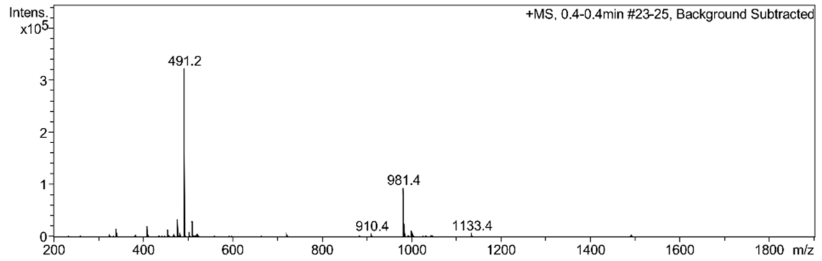

Synthetic information of peptide D206181

E

Peptide Name: Custom Peptide

Purchase order number: D206181

Lot Number: FL-10-0041

Sequence: H-Cys-Arg-Thr-Leu-Pro-Phe-Gly-Ser-Cys-OH (Cys&Cys bridge)

Molecular Weight (daltons) : 981.2

Final Purity (HPLC%) : 96.0%

Amount Supplied: 5mg

Appearance: White lyophilized powder

Storage Conditions: Long Term: -20°C; Short Term: 2°C ~ 8°C

Sample information

Sample Name: D206181/FL-10-0041
Sample Type : Unknown
Sample Bottle: 5
Number of Injections: 1
Injection Volume: 10.00 ul
Running Time: 20.0 Minutes

Collector: System
Sample Group Name: A20102701
Collection Method Group: 10 _ 40 B 07 _ method group
Approach: M20102702
Channel Name: DAD.0.0
Processing Channel Description: DAD: Signal A, 220nm/ broadband: 4nm

Acquisition Time: 2020/10/27 9:33:57 CST
Processing Time: 2020/10/27 9:58:37 CST

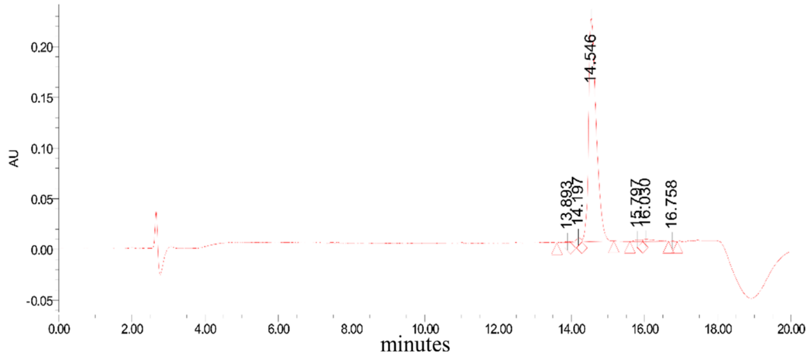

Peak Results

Name	Retention Time(minute)	%Area	Area(Microvolt*second)	Height(Microvolt)
1	13.893	0.35	11590	913
2	14.197	1.36	45358	4471
3	14.546	96.08	3214214	220311
4	15.797	0.74	24747	1771
5	16.030	1.39	46390	2145
6	16.758	0.10	3222	385

Mass Spectrum SmartFormula Report

Analysis Info

Analysis Name	D:\Data\ESI-LUH-201026-D206181_01.d	Acquisition Date	10/26/2020 3:58:52 PM
Method	lcms-5-55.m	Operator	BDAL@DE
Sample Name		Instrument / Ser#	microTOF-Q II 228888.10324
Comment			

Acquisition Parameter

Source Type	ESI	Ion Polarity	Positive	Set Nebulizer	1.8 Bar
Focus	Active	Set Capillary	4800 V	Set Dry Heater	220 °C
Scan Begin	100 m/z	Set End Plate Offset	-500 V	Set Dry Gas	6.0 l/min
Scan End	2000 m/z	Set Collision Cell RF	200.0 Vpp	Set Divert Valve	Waste

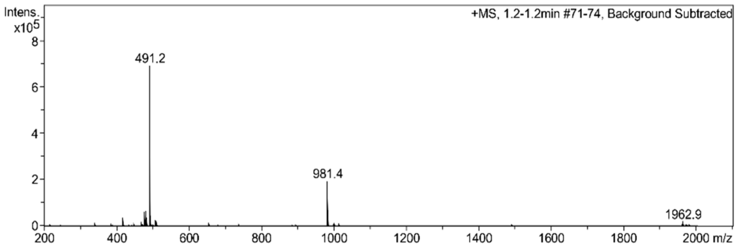

Figure S3. A) Synthetic information of peptide D206115. B) Synthetic information of peptide D206116. C) Synthetic information of peptide D206179. D) Synthetic information of peptide D206180. E) Synthetic information of peptide D206181.

Table S1. Data collection and refinement statistics.

	**WDR5-D206115**
**Wavelength**	
**Resolution range**	30.31-2.03 (2.103-2.03)
**Space group**	P 1
**Unit cell**	46.735 53.912 64.788 69.817 88.79 74.163
**Total reflections**	109572 (4876)
**Unique reflections**	32760 (1856)
**Multiplicity**	3.3 (2.6)
**Completeness (%)**	88.98 (50.71)
**Mean I/sigma(I)**	12.16 (2.05)
**Wilson B-factor**	28.18
**R-merge**	0.072 (0.4408)
**R-meas**	0.08559 (0.5513)
**R-pim**	0.04565 (0.3239)
**CC1/2**	0.998 (0.846)
**CC***	0.999 (0.957)
**Reflections used in refinement**	32718 (1855)
**Reflections used for R-free**	1621 (92)
**R-work**	0.1792 (0.2818)
**R-free**	0.2268 (0.3263)
**CC(work)**	0.968 (0.862)
**CC(free)**	0.946 (0.704)
**Number of non-hydrogen atoms**	5092

**macromolecules**	4786
**solvent**	306
**Protein residues**	618
**RMS(bonds)**	0.008
**RMS(angles)**	1.06
**Ramachandran favored (%)**	93.40
**Ramachandran allowed (%)**	6.11
**Ramachandran outliers (%)**	0.50
**Rotamer outliers (%)**	0.19
**Clashscore**	5.87
**Average B-factor**	32.80
**macromolecules**	32.55
**solvent**	36.64

Note：Statistics for the highest-resolution shell are shown in parentheses.

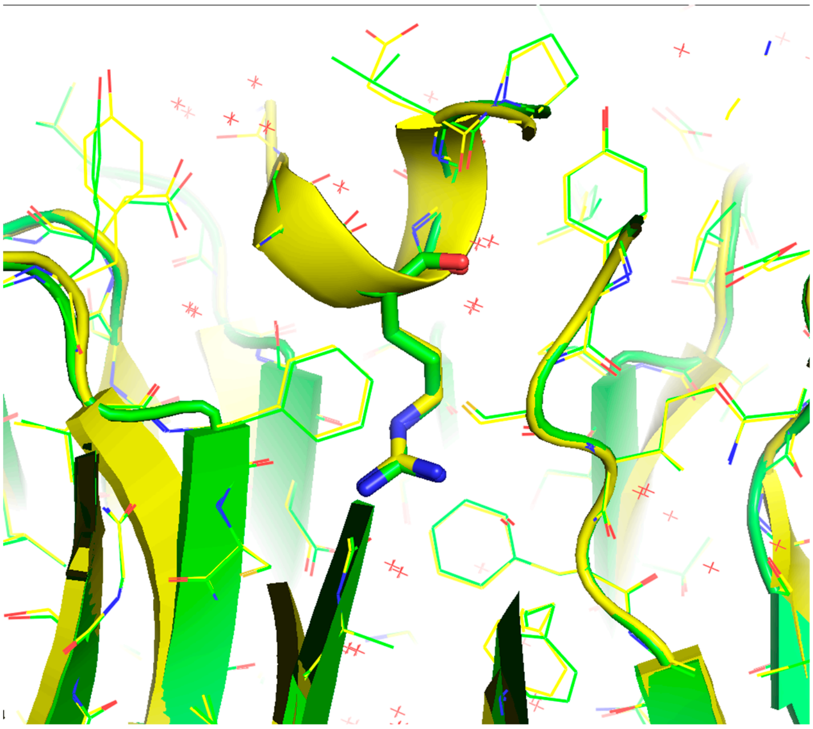

Figure S4. The comparison of cocrystal structure.
